# Sugar sensing and signaling in plants

**DOI:** 10.3389/fpls.2014.00113

**Published:** 2014-03-26

**Authors:** Sjef Smeekens, Hanjo A. Hellmann

**Affiliations:** ^1^Molecular Plant Physiology, Institute of Environmental Biology, Utrecht UniversityUtrecht, Netherlands; ^2^School of Biological Sciences, Washington State UniversityPullman, WA, USA

**Keywords:** sugar, signal transduction, sensing, metabolism, plant, transport, kinase

Sugars are ubiquitous and critical components for general metabolism. These primary products from photosynthesis affect most, if not all, processes in plant cells by providing skeletons for organic compounds and storing energy for chemical reactions. Sugars also serve as critical signaling molecules in relation to both cellular metabolic status and biotic and abiotic stress response (Rolland et al., 2006; Lastdrager et al., 2014). The diverse and complex networks sugars are involved in warrant a detailed comprehension of their impact on regulatory and metabolic processes at the cellular and the whole plant level. The current research topic on “Sugar Signaling and Sensing in Plants” is a combination of primary research articles and review work, and provide novel insights and detailed overviews on the current knowledge of sugars as metabolites and signal molecules.

The review article from Simone Ferrari and co-workers on oligogalacturonides (OGs) (Ferrari et al., 2013), illustrates an excellent example for a sugar being both a metabolite and a signaling molecule. OGs consist of α-1,4-linked galacturonosyl residues and are integral components of the cell wall. However upon biotic stresses, they can be released from the cell wall by hydrolytic enzymes activated by fungal growth or by mechanical damage inflicted through herbivores. Released OGs can then function as signaling molecules to elicit a defense response in the respective plant cell and surrounding tissues (Ferrari et al., 2013). Another aspect about sugars as signaling molecules is discussed in the article by Mohammad Bolouri Moghaddam and Wim Van den Ende that reports on the integration of sucrose-mediated signaling pathways in cellular networks. The paper discusses the interplay of sugar signals with other crucial cellular signaling systems, including the circadian clock and phytohormones, in controlling defense responses and developmental programs such as flowering (Bolouri Moghaddam and Van den Ende, 2013). Similarly, the regulatory steps that integrate diurnal signals with downstream cellular responses may occur at the sugar uptake step, which is indicated by the work from Chincinska and co-workers on the sucrose transporter 4 (SUT4) from potato (Chincinska et al., 2013). Another example of sugars functioning as regulatory molecules comes from the perspective article published by Dobrenel and co-workers on RAPAMYCIN (TOR) kinase complexes (Dobrenel et al., 2013). These complexes associate with additional partner proteins to affect and integrate a wide range of cellular responses, including metabolism, mRNA processing and autophagy, often in concert with nutrient signaling. Glucose has been reported as a positive regulator of TOR kinase activity and is discussed to affect diverse processes including biosynthesis of the stress-related sugar raffinose, glycolysis, and biosynthesis of sucrose and starch (Dobrenel et al., 2013).

Two facets of sugar biology that have been intensively investigated and that are characteristic for many sugars, especially sucrose, are their controlled subcellular distribution and long-distance transport from sinks to sources. Cellular sucrose metabolism depends on, and is limited by, the activities of sucrose synthase and sucrose-phosphate synthase (SPS). The work from Madoka Yonekura and co-workers provides new insights on two rice SPS paralogs, *OsSPS1* and *OsSPS11*, and their specific expressions in response to diurnal factors and carbohydrate availability (Yonekura et al., 2013). Sucrose long-distance transport is facilitated through the activities of specialized transport proteins. The work from Chincinska and co-workers investigates how these transporters may function as checkpoints to forward information on metabolic fluxes to initiate cellular responses (Chincinska et al., 2013).

Another centrally important question in sugar research is related to how sugars are perceived by the cell. The best evidence on cellular sugar sensing systems currently comes from hexose kinases, which phosphorylate glucose (hexokinase) and fructose (fructokinase). Hexokinase I from Arabidopsis has been implicated in these early steps (Jang et al., 1997; Moore et al., 2003), and two complementary overview articles in this research topic provide detailed updates on hexokinases and fructokinases in plants (Granot et al., 2013; Tiessen and Padilla-Chacon, 2013), as well as on other sugar metabolizing enzymes such as invertases, sucrose synthases, and SPS (Tiessen and Padilla-Chacon, 2013). These articles discuss knowledge that has been generated on the different proteins in context with their gene families, and on what is known about their subcellular localization and specific metabolic activities, as well as impacts on developmental programs and involvement in signal transduction events.

Additional regulatory steps in sugar and stress-related signal transduction depend mainly on the activity of SnRK1-protein kinases. These kinases are multi-subunit enzymes, to which cystathionine-β-synthase (CBS) domain-containing proteins belong. Interesting work from Timothy Heisel and co-workers shows that two of these subunits, AtPV42a and AtPV42b, are misregulated in histone acetyltransferase 1 (*hac1*) mutants (Heisel et al., 2013). *hac1* mutants show aberrant sugar-responses and fertility defects, which may in part be explained by the changed levels of *AtPV42a* and *AtPV42b* expression. In this context, the work from Ana Confraria and co-workers strongly implicate the participation of microRNAs in SnRK1-protein kinase-dependent processes (Confraria et al., 2013). Sugar response regulation also requires mRNA processing steps, as shown by Funck et al. (2012). Through map-based cloning of a sugar response mutation the authors identified ESP1, a CstF64-like putative RNA processing factor. ESP1 functions in mRNA 3′-end formation, and the work implicates RNA maturation as a critical factor for normal sugar response (Funck et al., 2012).

In summary the articles presented here emphasize the diversity of the processes that sugars are required for in the cell, and the regulatory networks in which they are involved. The articles also highlight the interplay between sugars and the circadian rhythm, and specific developmental programs, such as flowering. The broad-range of studies presented significantly deepen our knowledge about these important compounds, and demonstrate that, despite the already long-standing research on this topic, there remain many major issues that must be addressed if we are to understand the regulatory complexity and the components involved in sugar homeostasis, (sub)cellular allocation, and long-distance transport. Sugar signaling research will remain an exciting area of investigations for many years to come.
